# Kappa‐opioid receptors differentially regulate low and high levels of ethanol intake in female mice

**DOI:** 10.1002/brb3.523

**Published:** 2016-07-24

**Authors:** Ashlee Van't Veer, Karen L. Smith, Bruce M. Cohen, William A. Carlezon, Anita J. Bechtholt

**Affiliations:** ^1^ Department of PsychiatryHarvard Medical SchoolMcLean HospitalBelmontMAUSA; ^2^ National Institute of Neurological Disorders and StrokeNational Institutes of HealthBethesdaMDUSA; ^3^ National Institute on Alcohol Abuse and AlcoholismNational Institutes of HealthBethesdaMDUSA

**Keywords:** drinking, ethanol, kappa

## Abstract

**Introduction:**

Studies in laboratory animals and humans indicate that endogenous opioids play an important role in regulating the rewarding value of various drugs, including ethanol (EtOH). Indeed, opioid antagonists are currently a front‐line treatment for alcoholism in humans. Although roles for mu‐ and delta‐opioid receptors have been characterized, the contribution of kappa‐opioid receptors (KORs) is less clear. There is evidence that changes in KOR system function can decrease or increase EtOH drinking, depending on test conditions. For example, female mice lacking preprodynorphin – the precursor to the endogenous KOR ligand dynorphin – have reduced EtOH intake. Considering that KORs can regulate dopamine (DA) transmission, we hypothesized that KORs expressed on DA neurons would play a prominent role in EtOH intake in females.

**Methods:**

We used a Cre/*loxP* recombination strategy to ablate KORs throughout the body or specifically on dopamine uptake transporter (DAT)‐expressing neurons to investigate the role of KORs on preference for and intake of EtOH (2‐bottle choice), the transition from moderate to excessive EtOH drinking (intermittent EtOH access), and binge EtOH drinking (drinking in the dark [DID]).

**Results:**

KOR deletion decreased preference for EtOH, although this effect was less pronounced when EtOH intake increased beyond relatively low levels.

**Discussion:**

Our findings indicate that KOR activation increases EtOH drinking via effects mediated, at least in part, by KORs on DA neurons. While the mechanisms of this regulation remain unknown, previous work suggests that alterations in negative reinforcement processes or sensitivity to the sensory properties of EtOH can affect preference and intake.

## 1 Introduction

Endogenous opioids are involved in a variety of reward‐related behaviors. An important role for endogenous opioids in regulating the rewarding effects of ethanol (EtOH) has been established by findings using opioid antagonists in preclinical studies and by the effective clinical use of nonselective opioid antagonists in the treatment of alcoholism. One such antagonist, naltrexone, has been shown in numerous single center and multicenter placebo‐controlled clinical trials to improve treatment outcomes for alcoholics by decreasing relapse (Anton et al., 1999; Guardia et al., 2002; Heinala et al., 2001; Latt, Jurd, Houseman, & Wutzke, 2002; Oslin, Liberto, O'Brien, Krois, & Norbeck, 1997; Volpicelli, Alterman, Hayashida, & O'Brien, 1992), craving (Chick et al., 2000; Heinala et al., 2001; Volpicelli et al., 1992), days of drinking (Monti et al., 2001; O'Malley et al., 1992; Volpicelli et al., 1992) and number of drinks if the patient drank during treatment (Anton et al., 1999; Chick et al., 2000; Monti et al., 2001). Consistent with these clinical findings, there have also been reports from preclinical studies in laboratory animals that describe naltrexone‐induced decreases in ethanol intake (Froehlich, Harts, Lumeng, & Li, 1987, 1990; Hubbell et al., 1986; Myers & Lankford, 1996; Parkes & Sinclair, 2000; Phillips, Wenger, & Dorow, 1997; Reid & Hunter, 1984), ethanol self‐administration (Heyser, Roberts, Schulteis, & Koob, 1999; Samson & Doyle, 1985; Sinden, Marfaing‐Jallat, & Le Magnen, 1983; Williams, Kane, & Woods, 2001), and the expression of ethanol‐induced conditioned place preference (Bechtholt & Cunningham, 2005; Cunningham, Henderson, & Bormann, 1998; Kuzmin, Sandin, Terenius, & Ogren, 2003; Middaugh & Bandy, 2000). Nevertheless, naltrexone is not always effective in humans (Gastpar et al., 2002; Kranzler, Modesto‐Lowe, & Van Kirk, 2000; Krystal, Cramer, Krol, Kirk, & Rosenheck, 2001). The reasons for such variability are not known, but may be related to the fact that naltrexone has non‐selective actions as an antagonist at all opioid receptors, and broad blockade of opioid receptors can trigger aversive and/or depressive‐like signs (West & Wise, 1988). Such effects in humans might be expected to affect responsiveness to the medication as well as compliance to the treatment regimen.

It is important to determine whether the putative therapeutic effects of currently used opioid antagonists are attributable to actions on any specific receptor subtype, since selective antagonists are in various stages of development. Both mu‐opioid receptor (MOR) and delta‐opioid receptor (DOR) antagonists can selectively decrease EtOH consumption (Hyytia & Sinclair, 1993; Krishnan‐Sarin, Wand, Li, Portoghese, & Froehlich, 1998), suggesting that some of the effects of nonselective opioid antagonists may be mediated through these receptor systems. More recent work suggests that kappa‐opioid receptor (KOR) systems also influence EtOH intake, although these data include evidence that KOR activation can both increase and decrease EtOH drinking, depending on circumstances under which testing is performed. For example, KOR agonists decrease ethanol intake in rodents (Barson et al., 2010; Lindholm, Werme, Brene, & Franck, 2001; Nestby et al., 1999) and attenuate the function of midbrain (ventral tegmental area [VTA]) dopamine (DA) systems (Doyon, Howard, Shippenberg, & Gonzales, 2006; Lindholm et al., 2001), which are known to be critical mediators of natural and drug‐related rewards (Wise, 2013). However, KOR antagonists also reduce excessive alcohol consumption (Barson et al., 2010; Walker, Zorrilla, & Koob, 2011). Recent conceptualizations reconcile these seemingly disparate findings (i.e., KOR activation implicated in both increased and decreased drinking) by proposing that EtOH upregulates expression of dynorphin, an endogenous KOR ligand (Chavkin, James, & Goldstein, 1982) often associated with states of stress and dysphoria (see Bruchas, Land, & Chavkin, 2010; Van't Veer & Carlezon, 2013). According to this model (Kissler et al., 2014), the ability of EtOH to offset (via indirect actions mediated by DA) the aversive effects of elevated dynorphin actions at KORs enhances its ability to serve as a negative reinforcer (Wise & Koob, 2014) and thereby increases the motivation to drink. It is conceivable that gradual increases in endogenous dynorphin expression that occur as drinking behaviors become established have different consequences than acute administration of an exogenous KOR agonist once drinking behaviors are already established. Such findings are consistent with the considerable evidence that the motivational valence of KOR agonists are often different from – and in fact opposite to – those of MOR and DOR agonists (Van't Veer & Carlezon, 2013). KOR antagonists may decrease EtOH preference and intake by making it less effective as a negative reinforcer.

Studies of KOR antagonists are impeded by the unusual pharmacodynamics (i.e., slow onset, lack of initial selectivity for KORs, and exceptionally long duration of action) that are common to all the chemicals in this class that are broadly available at this time (Carroll & Carlezon, 2013). Genetic models have also been used to evaluate the role of KORs in EtOH drinking. Allelic variations in the KOR (*Oprk1*) and prodynorphin (*Pdyn*) genes are associated with alcohol dependence (Flory, Pytte, Hurd, Ferrell, & Manuck, 2011; Karpyak et al., 2013; Xuei et al., 2006), and constitutive KOR knockout mice exhibit a decrease in ethanol preference compared to wild‐type mice (Kovacs et al., 2005). Additionally, female (but not male) mice lacking preprodynorphin, the precursor for dynorphin, show decreases in preference and consumption of EtOH in a two‐bottle choice test compared to wild‐type littermates (Blednov, Walker, Martinez, & Harris, 2006). These data suggest that KOR activation can play an especially important role in EtOH drinking behavior in females. We hypothesized that studies of female mice in which KORs had been ablated from DA neurons may provide unique insights on the substrates of EtOH reward. Using a Cre/l*oxP* recombination system to create cell type specific KOR deletion (Van't Veer et al., 2013), we investigated the role of KORs in the expression of three types of ethanol consumption: (i) General stable EtOH preference and intake (2 bottle choice paradigm), (ii) escalation of drinking (intermittent EtOH access) and (iii) binge drinking (drinking in the dark [DID] paradigm). These procedures differentially provide metrics that reflect EtOH preference and intake patterns.

## 2 Methods

### 2.1 Mice

We used mutant mice in which exon 3 of the KOR gene (*Oprk1*) was flanked with loxP recombination sites, and bred them with lines expressing Cre‐recombinase (Cre) either in early embryogenesis (EIIa‐Cre) or only in dopamine (DA) neurons (dopamine transporter [DAT]‐Cre). This arrangement enabled us to develop separate lines of constitutive KOR knockouts (KOR^−/−^) and conditional knockouts that lack KORs in DA‐containing neurons (DAT‐KOR^lox/lox^). The development and validation of these mice has been described (Van't Veer et al., 2013). For the present experiments, KOR^−/−^ mice and littermate controls (KOR ^+/+^) were obtained by breeding KOR heterozygous (KOR^+/−^) mice. DAT‐ KOR^lox/lox^ and littermate controls (KOR^lox/lox^) were obtained by breeding floxed KOR mice expressing the DAT‐Cre transgene with floxed mice lacking the Cre transgene. Genomic DNA samples were obtained from tail biopsies and genotypes were determined by PCR as described in Van't Veer et al. (2013). Mice were backcrossed to C57BL/6J seven generations before testing. Experiments were conducted in female mice 2–4 months old at the start of each experiment. Separate cohorts of female mice (*n* = 7–11 per group) were used for each experiment. Female mice, which readily drink EtOH, were used to explore the potential role of KORs in light of evidence that problem drinking is becoming increasingly prevalent among women (White et al., 2015) and to complement and extend existing studies, many of which were conducted in males. Mice were housed individually and maintained on a 12:12 hr light–dark cycle with ad libitum food and water. In the two‐bottle choice experiment lights went on at 08:00. In the drinking in the dark and escalation experiments, the cycle was reversed such that lights went off at 08:00. Experimental protocols were approved by the McLean Hospital Institutional Animal Care and Use Committee in accordance with the National Institutes of Health policies.

### 2.2 Two bottle choice paradigm

This is a standard model used to generally quantify EtOH preference and intake in nondependent mice. Singly housed mice were given continuous (24 hr) access to tap water through two sipper tubes for 4 days (acclimation), after which one of the two water bottles was replaced with a bottle containing unsweetened EtOH in increasing concentrations (3%, 6%, and 10%, 15% v/v) available for 4 days each (Bechtholt, Smith, Raber, & Cunningham, 2004). To control for side bias, the left/right position of the bottles was reversed every 48 hr. Bottles were also cleaned at this time. Daily EtOH and water consumption (ml) were used to calculate EtOH intake (g kg^−1^) and preference ratio (EtOH intake/total intake).

### 2.3 Intermittent EtOH access

This model is used to study “loss of control” (e.g. the transition from moderate to excessive ethanol intake) but does not induce dependence. Intermittent EtOH (15% v/v) availability increases EtOH intake inducing an apparent “escalation” in EtOH intake (Melendez, 2011; Wise, 1973). Singly housed female mice were given access to tap water through two sipper tubes for 4 days (acclimation), after which one of the two water bottles was replaced with a bottle containing a 15% (v/v) unsweetened EtOH solution. Mice received access to EtOH every other day (i.e., 24 hr on, 24 hr off) made available 3 hr after the start of the dark phase of the light/dark cycle. To control for side bias, the left/right position of the bottles was reversed every 48 hr, at which time the used bottles were replaced with clean bottles. Daily EtOH and water consumption (ml) were used to calculate a total consumption, EtOH intake (g kg^−1^), and preference ratio (EtOH intake/total intake).

### 2.4 “Drinking in the dark” (DID) paradigm

The DID paradigm is a well‐validated behavioral model of nondependent excessive or “binge” EtOH intake (Rhodes, Best, Belknap, Finn, & Crabbe, 2005). Briefly, water bottles were replaced with 5 ml glass cylinders with stainless steel ball bearing sipper tubes attached with rubber septa. These drinking tubes were inserted through the bars of the wire cage tops and firmly attached using medium‐sized binder clips. Mice were given 2 hr access to an unsweetened 20% EtOH solution 3 hr into the dark phase of the light/dark cycle. To establish stable EtOH intakes mice were given EtOH access under these parameters for 5 days (pre‐exposure period) before testing. Volumes were recorded over a 4 hr test period. These procedures were modeled after (Kasten & Boehm, 2014).

### 2.5 Data analysis

Data are graphed as mean plus standard error of the mean. Data for each genotype were analyzed by two‐way ANOVA followed by Fisher's LSD or Dunnett's post hoc tests where appropriate.

## 3 Results

### 3.1 Two‐bottle choice paradigm

**Figure 1:**
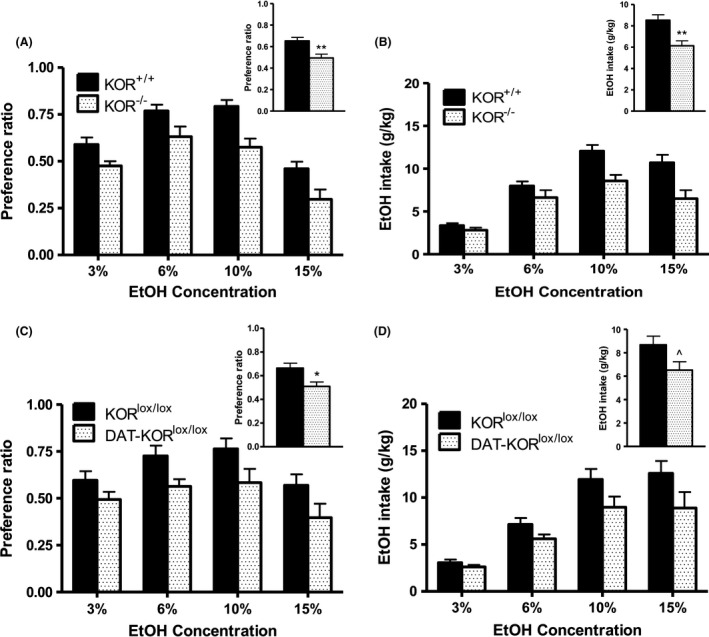
Ethanol preference in KOR ^−/−^ and DAT‐ KOR ^lox/lox^ mice compared to littermate controls as measured in the 2 bottle choice paradigm. Effect of KOR ^−/−^ on (A) preference ratio and (B) EtOH intake. Effect of DAT‐ KOR ^lox/lox^ on (C) preference ratio and (D) EtOH intake. Insets: Follow‐up comparisons between genotype were analyzed after collapsing across EtOH concentration. Data are expressed as means + *SEM* (*N* = 8–10 per group) Fisher's LSD post hoc analysis. *p < .05, **p < .01, ^*p* = .057

KOR^−/−^ mice expressed significantly lower preference for EtOH, as indicated by a significant main effect of Genotype (*F*_(1,18)_ = 10.71; *p* < .01; Fig. 1A inset). Preference ratio varied with EtOH concentration (main effect of EtOH concentration [*F*_(3,54)_ = 73.67; *p* < .0001]; Fig. 1A), but did not interact significantly with Genotype. Ethanol intake was also significantly lower in KOR^−/−^ mice compared to their KOR^+/+^ littermates as indicated by a significant main effect of Genotype (*F*_(1,18)_ = 11.66; *p* < .01; Fig. 1B inset). Ethanol intake increased with increasing EtOH concentrations (main effect of EtOH concentration [*F*_(3,54)_ = 58.33; *p* < .0001]); however, there was no interaction with Genotype.

Similarly, DAT‐KOR^lox/lox^ mice expressed significantly lower preference for EtOH, as indicated by a significant main effect of Genotype (*F*_(1,16)_ = 7.21; *p* < .05; Fig. 1C inset). A significant main effect of EtOH concentration was also observed (*F*_(3,48)_ = 7.15; *p* < .0001), with no interaction of factors (Fig. 1C). DAT‐KOR^lox/lox^ mice exhibited reduced EtOH intake, but this effect did not reach significance (*F*_(3,48)_ = 4.22; *p* = .057; Fig. 1D inset). A main effect of EtOH concentration (*F*_(3,48)_ = 46.31; *p* < .0001), but no interaction of factors, was detected (Fig. 1D).

### 3.2 Effect of KOR in escalated EtOH drinking in the intermittent access paradigm

**Figure 2:**
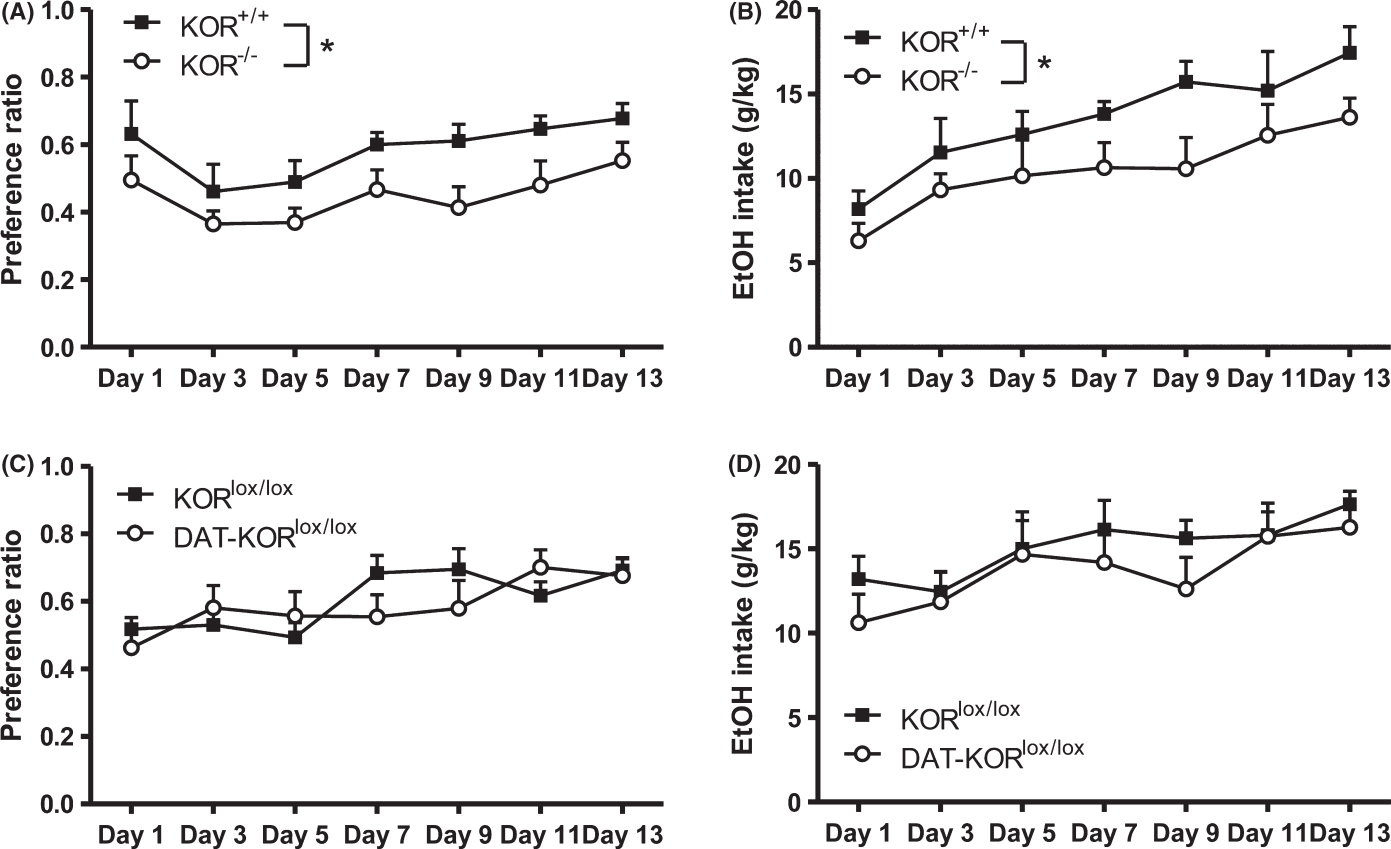
Escalation of drinking in KOR ^−/−^ and DAT‐ KOR ^lox/lox^ compared to littermate controls as measured in the intermittent access paradigm. Effect of KOR ^−/−^ on (A) preference ratio and (B) EtOH intake. Follow‐up comparisons between genotype were analyzed after collapsing across Day for preference ratio and EtOH intake. DAT‐ KOR ^lox/lox^ mice were not significantly different from KOR ^lox/lox^ controls in (C) preference ratio or (D) EtOH intake. Data are expressed as means + *SEM* (*N* = 7–11 per group) Fisher's LSD post hoc analysis. *p < .05

Preference ratio was significantly lower in KOR^−/−^ mice compared to littermate controls in the intermittent access paradigm (main effect of Genotype [*F*_(1,13)_ = 5.70; *p* < .05; Fig. 2A]). KOR^−/−^ mice also expressed significantly lower EtOH intake as indicated by a significant main effect of Genotype (*F*_(1,13)_ = 6.95; *p* < .05; Fig. 2B). Both preference ratio and EtOH intake increased across days (*F*_(6,78)_ = 4.88; *p* < .001, *F*_(6,78)_ = 7.00; *p* < .0001, respectively) but were not dependent on Genotype (Fig. 2A and B). Follow‐up tests on EtOH intake collapsed across genotype demonstrated a significant difference between Day 1 and all other days (data not shown), indicating mice escalated their intake during intermittent access, as expected. While preference ratio and EtOH intake increased across days (*F*_(6,102)_ = 5.85; *p* < .0001, *F*_(6,102)_ = 5.04; *p* < .0001, respectively), no significant effects were detected between DAT‐KOR^lox/lox^ mice and their littermate controls in the intermittent access paradigm (Fig. 2C and D).

### 3.3 Effect of KOR on binge drinking in the DID paradigm

**Figure 3:**
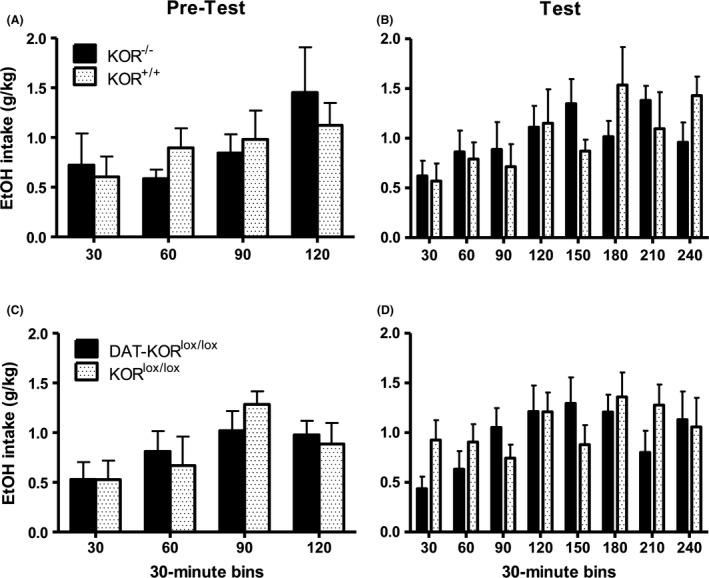
Binge drinking in KOR ^−/−^ and DAT‐ KOR ^lox/lox^ mice compared to controls in the DID paradigm. Effect of KOR ^−/−^ compared to KOR ^+/+^ controls on EtOH intake during the final 2‐hr pretest (A) and 4‐hr test (B). Effect of DAT‐ KOR ^lox/lox^ compared to KOR ^lox/lox^ controls on EtOH intake during the final 2‐hr pretest (C) and 4‐hr test (D). Knockout mice were not significantly different from their respective littermate controls. Data are expressed as means + *SEM* (*N* = 6–10 per group)

No statistical differences were detected between either KOR mutant line compared to their respective littermate controls in binge EtOH drinking (DID) in either the final 2‐hr pretest (Fig. 3A and C) or the 4‐hr test (Fig. 3B and D). Data were collected in 30 min intervals for the duration of the test. During the final pretest, a significant effect of Time emerged in the collapsed data for DAT‐ KOR^lox/lox^ and KOR^lox/lox^ (*F*_(3,48)_ = 3.11; *p* < .05) revealing a significant difference between the 30 and 90 min bin (Fig. 3C). No effects of Genotype or interactions were detected. Similarly, during the test session, a significant effect of Time emerged in the collapsed data for KOR^−/−^ and KOR^+/+^ groups (*F*_(7,98)_ = 2.15; *p* < .05); however, follow‐up analyses found no significant differences among 30 min time bins (Fig. 3B). No effects of Time, Genotype, or interactions were detected in the DAT‐KOR^lox/lox^ experiment.

## 4 Discussion

Here, we examined the role of KORs in EtOH drinking in females, using two lines of mutant (knock out) KOR mice in paradigms designed to model general preference for and intake of EtOH (2 bottle choice paradigm), escalation of EtOH drinking (intermittent EtOH access), and binge EtOH drinking (drinking in the dark [DID] paradigm) without inducing dependence. Both the constitutive knockout line (KOR^−/−^), in which KORs are ablated throughout the body, and our conditional line (DAT‐KOR^lox/lox^), in which KORs are ablated in DAT‐expressing cells only, exhibited significant decreases in EtOH preference in a two‐bottle choice paradigm. In addition, EtOH intake was significantly decreased in KOR^−/−^ mice and approached significance in DAT‐KOR^lox/lox^ mice compared to littermate control mice. These data indicate that EtOH preference and intake are regulated, at least in part, by KORs on DAT‐expressing neurons such as those that project from the ventral tegmental area (VTA) to the nucleus accumbens (NAcc). Acquisition of moderate‐to‐excessive EtOH consumption was also attenuated in KOR^−/−^ mice, whereas DAT‐KOR^lox/lox^ were indistinguishable from littermate controls. KOR ablation did not reduce binge drinking in either knockout line. Considered together, these findings suggest that KORs in regions expressing DAT may mediate vulnerability to low and moderate intake, but play a lesser role in the regulation of excessive EtOH intake.

The binding of the endogenous ligand dynorphin to KORs can induce aversive or depressive‐like states, which can be quantified in rodents using procedures such as intracranial self‐stimulation (ICSS; Todtenkopf, Marcus, Portoghese, & Carlezon, 2004). This effect is thought to be mediated by decreased DA neurotransmission in the VTA and NAcc (Di Chiara & Imperato, 1988; Koob, 1992). This inhibition may serve a counter or regulatory role to the actions of endorphin and enkephalin at mu and delta opioid receptors, respectively, which facilitate dopamine release (Devine, Leone, Pocock, & Wise, 1993) and activate processes associated with reward and reinforcement (Wise & Rompre, 1989). The ability to elevate brain DA function has been implicated in the rewarding effects of a wide variety of abused drugs, including EtOH (see Koob & Weiss, 1992; Wise & Bozarth, 1987). According to this model, removal of KOR‐mediated inhibition through pharmacologic blockade or gene knockout might be expected to facilitate EtOH reward and increase intake, as has been reported after administration of the KOR antagonist norBNI (Mitchell, Liang, & Fields, 2005). Here, however, we report a significant decrease in intake in mice lacking KORs, an effect consistent with previous findings using similar genetic models, where both EtOH and sucrose intake were reduced in mice lacking preprodynorphin (Blednov et al., 2006) or KORs (Kovacs et al., 2005). The corresponding decreases in EtOH preference observed here is more suggestive of a decreases in EtOH reward in the absence of KORs.

One possible explanation for our findings is that ablation of KORs could alter sensory functions that contribute to intake. Previous findings in mice lacking KORs or preprodynorphin have yielded similar results and also demonstrated a decrease in intake of sucrose together with increased quinine intake (Kovacs et al., 2005). These findings led to the speculation that a lack of KOR activation or an associated compensatory response to KOR knockout altered the tastant response or orosensory reward. Indeed, a palatable diet increases hypothalamic dynorphin peptide and mRNA levels (Welch, Kim, Grace, Billington, & Levine, 1996). In the present work we show decreased EtOH intake in mice lacking KORs specifically in DAT‐expressing neurons. Given that palatable tastes stimulate DA release (Ahn & Phillips, 1999) and DA depletion of the VTA inhibits consumption (Martinez‐Hernandez, Lanuza, & Martinez‐Garcia, 2006; Shimura, Kamada, & Yamamoto, 2002), we would expect to observe increases in EtOH intake in the conditional knockout because KOR ablation is predicted to increase DA neurotransmission. Because we only examined the effects of KOR deletion in models of ethanol consumption, it is not clear whether these effects are specific to ethanol consumption or would generalize to other reinforcers.

Ablation of KORs may also weaken a negative reinforcement process that can affect the intake of EtOH and other drugs (Markou, Kosten, & Koob, 1998; Wise & Koob, 2014). It has been recently proposed that EtOH intake elevates dynorphin expression, which elicits states of dysphoria or anxiety that are mitigated by further EtOH intake (Kissler et al., 2014). While our results seem broadly consistent with the ability of EtOH to serve as a negative reinforcer, in that reductions in KOR function would be expected to be accompanied by reductions in aversive states that are mitigated by EtOH intake, there are some potential inconsistencies that remain to be reconciled. Foremost, at least in male rodents, the ability of KOR antagonists to reduce EtOH reward reportedly occurs only in dependent animals (Kissler et al., 2014). The present data indicate that the effects of KOR deletion on EtOH consumption are observed in the two‐bottle choice paradigm, in which intakes are relatively low and animals are nondependent. Additionally, it is important to emphasize that KOR deletion did not reduce EtOH intake in all paradigms we used. Escalation of EtOH intake in the intermittent EtOH access paradigm was reduced in KOR^−/−^ mice but not DAT‐KOR^lox/lox^ mice, whereas KOR deletion had no effect in either genetic model when the mice were tested in the drinking in the dark, (binge drinking) paradigm. It is known that a different combination of genes underlie these different types of ethanol intake (Rosenwasser, Fixaris, Crabbe, Brooks, & Ascheid, 2013), suggesting disparate mechanisms. Thus it is possible that the mechanisms leading to a decrease in EtOH intake resulting from KOR deletion may not be relevant in a binge drinking paradigm. Also, while KOR antagonism does not reduce ethanol drinking in relatively low‐drinking nondependent rats (e.g. Walker et al., 2011), KOR deletion does appear reduce ethanol drinking in low‐drinking two‐bottle choice mice. This apparent discrepancy could be explained by species differences relating to motivation to drink (e.g., taste) or developmental compensation. Additional research beyond the scope of this work may clarify mechanistic differences in each paradigm, and help to resolve the differential responses observed here.

In summary, our findings show that both constitutive and conditional female KOR knockout mice demonstrate decreased ethanol intake and preference, suggesting that KORs can regulate EtOH drinking when intakes are low. We have also shown that both constitutive and DAT‐containing neuron‐specific ablation of KORs had no effect on excessive EtOH intake as measured by the drinking in the dark paradigm. Our findings contribute to a growing literature regarding the role of KORs in ethanol reward and suggest that the role of KORs is dependent upon the level of EtOH intake.

## Funding Information

Funding for this work was provided by NIH (R03AA019577 to AJB; MH063266 to WAC), a National Defense Science and Engineering Graduate Fellowship (to AVV), the Shervert Frazier Research Institute (to BMC), and the Englehard Foundation (to BMC).

## Conflict of Interest

None declared.

## References

[brb3523-bib-0001] Ahn, S. , & Phillips, A. G. (1999). Dopaminergic correlates of sensory‐specific satiety in the medial prefrontal cortex and nucleus accumbens of the rat. The Journal of Neuroscience, 19, RC29. 10493774 10.1523/JNEUROSCI.19-19-j0003.1999 PMC6782999

[brb3523-bib-0002] Anton, R. F. , Moak, D. H. , Waid, L. R. , Latham, P. K. , Malcolm, R. J. , & Dias, J. K. (1999). Naltrexone and cognitive behavioral therapy for the treatment of outpatient alcoholics: Results of a placebo‐controlled trial. The American Journal of Psychiatry, 156, 1758–1764. 10553740 10.1176/ajp.156.11.1758

[brb3523-bib-0003] Barson, J. R. , Carr, A. J. , Soun, J. E. , Sobhani, N. C. , Rada, P. , Leibowitz, S. F. , & Hoebel, B. G. (2010). Opioids in the hypothalamic paraventricular nucleus stimulate ethanol intake. Alcoholism, Clinical and Experimental Research, 34, 214–222. 10.1111/j.1530-0277.2009.01084.x PMC5266508 19951300

[brb3523-bib-0004] Bechtholt, A. J. , & Cunningham, C. L. (2005). Ethanol‐induced conditioned place preference is expressed through a ventral tegmental area dependent mechanism. Behavioral Neuroscience, 119, 213–223. 15727526 10.1037/0735-7044.119.1.213

[brb3523-bib-0005] Bechtholt, A. J. , Smith, R. , Raber, J. , & Cunningham, C. L. (2004). Enhanced ethanol‐, but not cocaine‐induced, conditioned place preference in Apoe(−/−) mice. Pharmacology, Biochemistry, and Behavior, 77, 783–792. 10.1016/j.pbb.2004.02.002 15099924

[brb3523-bib-0006] Blednov, Y. A. , Walker, D. , Martinez, M. , & Harris, R. A. (2006). Reduced alcohol consumption in mice lacking preprodynorphin. Alcohol, 40, 73–86. 17307643 10.1016/j.alcohol.2006.12.002 PMC1850187

[brb3523-bib-0007] Bruchas, M. R. , Land, B. B. , & Chavkin, C. (2010). The dynorphin/kappa opioid system as a modulator of stress‐induced and pro‐addictive behaviors. Brain Research, 1314, 44–55. 19716811 10.1016/j.brainres.2009.08.062 PMC2819621

[brb3523-bib-0008] Carroll, F. I. , & Carlezon, W. A. Jr (2013). Development of kappa opioid receptor antagonists. Journal of Medicinal Chemistry, 56, 2178–2195. 23360448 10.1021/jm301783x PMC3612131

[brb3523-bib-0009] Chavkin, C. , James, I. F. , & Goldstein, A. (1982). Dynorphin is a specific endogenous ligand of the kappa opioid receptor. Science, 215, 413–415. 6120570 10.1126/science.6120570

[brb3523-bib-0010] Chick, J. , Anton, R. , Checinski, K. , Croop, R. , Drummond, D. C. , Farmer, R. , … Ritson, B. (2000). A multicentre, randomized, double‐blind, placebo‐controlled trial of naltrexone in the treatment of alcohol dependence or abuse. Alcohol and Alcoholism, 35, 587–593. 11093966 10.1093/alcalc/35.6.587

[brb3523-bib-0011] Cunningham, C. L. , Henderson, C. M. , & Bormann, N. M. (1998). Extinction of ethanol‐induced conditioned place preference and conditioned place aversion: Effects of naloxone. Psychopharmacology (Berl), 139, 62–70. 9768543 10.1007/s002130050690

[brb3523-bib-0012] Devine, D. P. , Leone, P. , Pocock, D. , & Wise, R. A. (1993). Differential involvement of ventral tegmental mu, delta and kappa opioid receptors in modulation of basal mesolimbic dopamine release: In vivo microdialysis studies. The Journal of Pharmacology and Experimental Therapeutics, 266, 1236–1246. 7690399

[brb3523-bib-0013] Di Chiara, G. , & Imperato, A. (1988). Opposite effects of mu and kappa opiate agonists on dopamine release in the nucleus accumbens and in the dorsal caudate of freely moving rats. The Journal of Pharmacology and Experimental Therapeutics, 244, 1067–1080. 2855239

[brb3523-bib-0014] Doyon, W. M. , Howard, E. C. , Shippenberg, T. S. , & Gonzales, R. A. (2006). Kappa‐opioid receptor modulation of accumbal dopamine concentration during operant ethanol self‐administration. Neuropharmacology, 51, 487–496. 16781738 10.1016/j.neuropharm.2006.04.005 PMC1973091

[brb3523-bib-0015] Flory, J. D. , Pytte, C. L. , Hurd, Y. , Ferrell, R. E. , & Manuck, S. B. (2011). Alcohol dependence, disinhibited behavior and variation in the prodynorphin gene. Biological Psychology, 88, 51–56. 21736916 10.1016/j.biopsycho.2011.06.007 PMC3171516

[brb3523-bib-0016] Froehlich, J. C. , Harts, J. , Lumeng, L. , & Li, T. K. (1987). Naloxone attenuation of voluntary alcohol consumption. Alcohol and Alcoholism, (Suppl 1), 333–337. 3426696

[brb3523-bib-0017] Froehlich, J. C. , Harts, J. , Lumeng, L. , & Li, T. K. (1990). Naloxone attenuates voluntary ethanol intake in rats selectively bred for high ethanol preference. Pharmacology, Biochemistry, and Behavior, 35, 385–390. 10.1016/0091-3057(90)90174-g 2320646

[brb3523-bib-0018] Gastpar, M. , Bonnet, U. , Boning, J. , Mann, K. , Schmidt, L. G. , Soyka, M. , … Croop, R. (2002). Lack of efficacy of naltrexone in the prevention of alcohol relapse: Results from a German multicenter study. Journal of Clinical Psychopharmacology, 22, 592–598. 12454559 10.1097/00004714-200212000-00009

[brb3523-bib-0019] Guardia, J. , Caso, C. , Arias, F. , Gual, A. , Sanahuja, J. , Ramirez, M. , … Casas, M. (2002). A double‐blind, placebo‐controlled study of naltrexone in the treatment of alcohol‐dependence disorder: Results from a multicenter clinical trial. Alcoholism, Clinical and Experimental Research, 26, 1381–1387. 10.1097/01.ALC.0000030561.15921.A9 12351933

[brb3523-bib-0020] Heinala, P. , Alho, H. , Kiianmaa, K. , Lonnqvist, J. , Kuoppasalmi, K. , & Sinclair, J. D. (2001). Targeted use of naltrexone without prior detoxification in the treatment of alcohol dependence: A factorial double‐blind, placebo‐controlled trial. Journal of Clinical Psychopharmacology, 21, 287–292. 11386491 10.1097/00004714-200106000-00006

[brb3523-bib-0021] Heyser, C. J. , Roberts, A. J. , Schulteis, G. , & Koob, G. F. (1999). Central administration of an opiate antagonist decreases oral ethanol self‐administration in rats. Alcoholism, Clinical and Experimental Research, 23, 1468–1476. 10512312

[brb3523-bib-0022] Hubbell, C. L. , Czirr, S. A. , Hunter, G. A. , Beaman, C. M. , LeCann, N. C. , & Reid, L. D. (1986). Consumption of ethanol solution is potentiated by morphine and attenuated by naloxone persistently across repeated daily administrations. Alcohol, 3, 39–54. 3964437 10.1016/0741-8329(86)90070-4

[brb3523-bib-0023] Hyytia, P. , & Sinclair, J. D. (1993). Responding for oral ethanol after naloxone treatment by alcohol‐preferring AA rats. Alcoholism, Clinical and Experimental Research, 17, 631–636. 10.1111/j.1530-0277.1993.tb00810.x 8392818

[brb3523-bib-0024] Karpyak, V. M. , Winham, S. J. , Preuss, U. W. , Zill, P. , Cunningham, J. M. , Walker, D. L. , … Biernacka, J. M. (2013). Association of the PDYN gene with alcohol dependence and the propensity to drink in negative emotional states. International Journal of Neuropsychopharmacology, 16, 975–985. 23101464 10.1017/S1461145712001137 PMC3901318

[brb3523-bib-0025] Kasten, C. R. , & Boehm, S. L. 2nd (2014). Intra‐nucleus accumbens shell injections of R(+)‐ and S(−)‐baclofen bidirectionally alter binge‐like ethanol, but not saccharin, intake in C57Bl/6J mice. Behavioural Brain Research, 272, 238–247. 25026094 10.1016/j.bbr.2014.07.011 PMC4134668

[brb3523-bib-0026] Kissler, J. L. , Sirohi, S. , Reis, D. J. , Jansen, H. T. , Quock, R. M. , Smith, D. G. , & Walker, B. M. (2014). The one‐two punch of alcoholism: Role of central amygdala dynorphins/kappa‐opioid receptors. Biological Psychiatry, 75, 774–782. 23611261 10.1016/j.biopsych.2013.03.014 PMC3749293

[brb3523-bib-0027] Koob, G. F. (1992). Neural mechanisms of drug reinforcement. Annals of the New York Academy of Sciences, 654, 171–191. 1632582 10.1111/j.1749-6632.1992.tb25966.x

[brb3523-bib-0028] Koob, G. F. , & Weiss, F. (1992). Neuropharmacology of cocaine and ethanol dependence. Recent Developments in Alcoholism, 10, 201–233. 1350359 10.1007/978-1-4899-1648-8_11

[brb3523-bib-0029] Kovacs, K. M. , Szakall, I. , O'Brien, D. , Wang, R. , Vinod, K. Y. , Saito, M. , … Vadasz, C. (2005). Decreased oral self‐administration of alcohol in kappa‐opioid receptor knock‐out mice. Alcoholism, Clinical and Experimental Research, 29, 730–738. 10.1097/01.alc.0000164361.62346.d6 15897716

[brb3523-bib-0030] Kranzler, H. R. , Modesto‐Lowe, V. , & Van Kirk, J. (2000). Naltrexone vs. nefazodone for treatment of alcohol dependence. A placebo‐controlled trial. Neuropsychopharmacology, 22, 493–503. 10731624 10.1016/S0893-133X(99)00135-9

[brb3523-bib-0031] Krishnan‐Sarin, S. , Wand, G. S. , Li, X. W. , Portoghese, P. S. , & Froehlich, J. C. (1998). Effect of mu opioid receptor blockade on alcohol intake in rats bred for high alcohol drinking. Pharmacology, Biochemistry, and Behavior, 59, 627–635. 10.1016/s0091-3057(97)00474-7 9512064

[brb3523-bib-0032] Krystal, J. H. , Cramer, J. A. , Krol, W. F. , Kirk, G. F. , & Rosenheck, R. A. (2001). Naltrexone in the treatment of alcohol dependence. New England Journal of Medicine, 345, 1734–1739. 11742047 10.1056/NEJMoa011127

[brb3523-bib-0033] Kuzmin, A. , Sandin, J. , Terenius, L. , & Ogren, S. O. (2003). Acquisition, expression, and reinstatement of ethanol‐induced conditioned place preference in mice: Effects of opioid receptor‐like 1 receptor agonists and naloxone. The Journal of Pharmacology and Experimental Therapeutics, 304, 310–318. 12490606 10.1124/jpet.102.041350

[brb3523-bib-0034] Latt, N. C. , Jurd, S. , Houseman, J. , & Wutzke, S. E. (2002). Naltrexone in alcohol dependence: A randomised controlled trial of effectiveness in a standard clinical setting. Medical Journal of Australia, 176, 530–534. 12064984 10.5694/j.1326-5377.2002.tb04550.x

[brb3523-bib-0035] Lindholm, S. , Werme, M. , Brene, S. , & Franck, J. (2001). The selective kappa‐opioid receptor agonist U50,488H attenuates voluntary ethanol intake in the rat. Behavioural Brain Research, 120, 137–146. 11182162 10.1016/s0166-4328(00)00368-5

[brb3523-bib-0036] Markou, A. , Kosten, T. R. , & Koob, G. F. (1998). Neurobiological similarities in depression and drug dependence: A self‐medication hypothesis. Neuropsychopharmacology, 18, 135–174. 9471114 10.1016/S0893-133X(97)00113-9

[brb3523-bib-0037] Martinez‐Hernandez, J. , Lanuza, E. , & Martinez‐Garcia, F. (2006). Selective dopaminergic lesions of the ventral tegmental area impair preference for sucrose but not for male sexual pheromones in female mice. The European Journal of Neuroscience, 24, 885–893. 16930416 10.1111/j.1460-9568.2006.04944.x

[brb3523-bib-0038] Melendez, R. I. (2011). Intermittent (every‐other‐day) drinking induces rapid escalation of ethanol intake and preference in adolescent and adult C57BL/6J mice. Alcoholism, Clinical and Experimental Research, 35, 652–658. 10.1111/j.1530-0277.2010.01383.x PMC3066271 21223302

[brb3523-bib-0039] Middaugh, L. D. , & Bandy, A. L. (2000). Naltrexone effects on ethanol consumption and response to ethanol conditioned cues in C57BL/6 mice. Psychopharmacology (Berl), 151, 321–327. 11026738 10.1007/s002130000479

[brb3523-bib-0040] Mitchell, J. M. , Liang, M. T. , & Fields, H. L. (2005). A single injection of the kappa opioid antagonist norbinaltorphimine increases ethanol consumption in rats. Psychopharmacology (Berl), 182, 384–392. 16001119 10.1007/s00213-005-0067-7

[brb3523-bib-0041] Monti, P. M. , Rohsenow, D. J. , Swift, R. M. , Gulliver, S. B. , Colby, S. M. , Mueller, T. I. , … Asher, M. K. (2001). Naltrexone and cue exposure with coping and communication skills training for alcoholics: Treatment process and 1‐year outcomes. Alcoholism, Clinical and Experimental Research, 25, 1634–1647. 11707638

[brb3523-bib-0042] Myers, R. D. , & Lankford, M. F. (1996). Suppression of alcohol preference in high alcohol drinking rats: Efficacy of amperozide versus naltrexone. Neuropsychopharmacology, 14, 139–149. 8822536 10.1016/0893-133X(95)00081-N

[brb3523-bib-0043] Nestby, P. , Schoffelmeer, A. N. , Homberg, J. R. , Wardeh, G. , De Vries, T. J. , Mulder, A. H. , & Vanderschuren, L. J. (1999). Bremazocine reduces unrestricted free‐choice ethanol self‐administration in rats without affecting sucrose preference. Psychopharmacology (Berl), 142, 309–317. 10208324 10.1007/s002130050894

[brb3523-bib-0044] O'Malley, S. S. , Jaffe, A. J. , Chang, G. , Schottenfeld, R. S. , Meyer, R. E. , & Rounsaville, B. (1992). Naltrexone and coping skills therapy for alcohol dependence. A controlled study. Archives of General Psychiatry, 49, 881–887. 1444726 10.1001/archpsyc.1992.01820110045007

[brb3523-bib-0045] Oslin, D. , Liberto, J. G. , O'Brien, J. , Krois, S. , & Norbeck, J. (1997). Naltrexone as an adjunctive treatment for older patients with alcohol dependence. The American Journal of Geriatric Psychiatry, 5, 324–332. 9363289 10.1097/00019442-199700540-00007

[brb3523-bib-0046] Parkes, H. , & Sinclair, J. D. (2000). Reduction of alcohol drinking and upregulation of opioid receptors by oral naltrexone in AA rats. Alcohol, 21, 215–221. 11091024 10.1016/s0741-8329(00)00091-4

[brb3523-bib-0047] Phillips, T. J. , Wenger, C. D. , & Dorow, J. D. (1997). Naltrexone effects on ethanol drinking acquisition and on established ethanol consumption in C57BL/6J mice. Alcoholism, Clinical and Experimental Research, 21, 691–702. 9194926

[brb3523-bib-0048] Reid, L. D. , & Hunter, G. A. (1984). Morphine and naloxone modulate intake of ethanol. Alcohol, 1, 33–37. 6543609 10.1016/0741-8329(84)90033-8

[brb3523-bib-0049] Rhodes, J. S. , Best, K. , Belknap, J. K. , Finn, D. A. , & Crabbe, J. C. (2005). Evaluation of a simple model of ethanol drinking to intoxication in C57BL/6J mice. Physiology & Behavior, 84, 53–63. 15642607 10.1016/j.physbeh.2004.10.007

[brb3523-bib-0050] Rosenwasser, A. M. , Fixaris, M. C. , Crabbe, J. C. , Brooks, P. C. , & Ascheid, S. (2013). Escalation of intake under intermittent ethanol access in diverse mouse genotypes. Addiction Biology, 18, 496–507. 22862671 10.1111/j.1369-1600.2012.00481.x PMC3508291

[brb3523-bib-0051] Samson, H. H. , & Doyle, T. F. (1985). Oral ethanol self‐administration in the rat: Effect of naloxone. Pharmacology, Biochemistry, and Behavior, 22, 91–99. 10.1016/0091-3057(85)90491-5 3975250

[brb3523-bib-0052] Shimura, T. , Kamada, Y. , & Yamamoto, T. (2002). Ventral tegmental lesions reduce overconsumption of normally preferred taste fluid in rats. Behavioural Brain Research, 134, 123–130. 12191798 10.1016/s0166-4328(01)00461-2

[brb3523-bib-0053] Sinden, J. D. , Marfaing‐Jallat, P. , & Le Magnen, J. (1983). The effect of naloxone on intragastric ethanol self‐administration. Pharmacology, Biochemistry, and Behavior, 19, 1045–1048. 10.1016/0091-3057(83)90414-8 6657723

[brb3523-bib-0054] Todtenkopf, M. S. , Marcus, J. F. , Portoghese, P. S. , & Carlezon, W. A. Jr (2004). Effects of kappa‐opioid receptor ligands on intracranial self‐stimulation in rats. Psychopharmacology (Berl), 172, 463–470. 14727002 10.1007/s00213-003-1680-y

[brb3523-bib-0055] Van't Veer, A. , Bechtholt, A. J. , Onvani, S. , Potter, D. , Wang, Y. , Liu‐Chen, L. Y. , … Carlezon, W. A. Jr (2013). Ablation of kappa‐opioid receptors from brain dopamine neurons has anxiolytic‐like effects and enhances cocaine‐induced plasticity. Neuropsychopharmacology, 38, 1585–1597. 23446450 10.1038/npp.2013.58 PMC3682153

[brb3523-bib-0056] Van't Veer, A. , & Carlezon, W. A. Jr (2013). Role of kappa‐opioid receptors in stress and anxiety‐related behavior. Psychopharmacology (Berl), 229, 435–452. 23836029 10.1007/s00213-013-3195-5 PMC3770816

[brb3523-bib-0057] Volpicelli, J. R. , Alterman, A. I. , Hayashida, M. , & O'Brien, C. P. (1992). Naltrexone in the treatment of alcohol dependence. Archives of General Psychiatry, 49, 876–880. 1345133 10.1001/archpsyc.1992.01820110040006

[brb3523-bib-0058] Walker, B. M. , Zorrilla, E. P. , & Koob, G. F. (2011). Systemic kappa‐opioid receptor antagonism by nor‐binaltorphimine reduces dependence‐induced excessive alcohol self‐administration in rats. Addiction Biology, 16, 116–119. 20579007 10.1111/j.1369-1600.2010.00226.x PMC2988980

[brb3523-bib-0059] Welch, C. C. , Kim, E. M. , Grace, M. K. , Billington, C. J. , & Levine, A. S. (1996). Palatability‐induced hyperphagia increases hypothalamic Dynorphin peptide and mRNA levels. Brain Research, 721, 126–131. 8793092 10.1016/0006-8993(96)00151-5

[brb3523-bib-0060] West, T. E. , & Wise, R. A. (1988). Effects of naltrexone on nucleus accumbens, lateral hypothalamic and ventral tegmental self‐stimulation rate‐frequency functions. Brain Research, 462, 126–133. 3179728 10.1016/0006-8993(88)90594-x

[brb3523-bib-0061] White, A. , Castle, I. J. , Chen, C. M. , Shirley, M. , Roach, D. , & Hingson, R. (2015). Converging patterns of alcohol use and related outcomes among females and males in the United States, 2002 to 2012. Alcoholism, Clinical and Experimental Research, 39, 1712–1726. 10.1111/acer.12815 26331879

[brb3523-bib-0062] Williams, K. L. , Kane, E. C. , & Woods, J. H. (2001). Interaction of morphine and naltrexone on oral ethanol self‐administration in rhesus monkeys. Behavioural Pharmacology, 12, 325–333. 11710747 10.1097/00008877-200109000-00003

[brb3523-bib-0063] Wise, R. A. (1973). Voluntary ethanol intake in rats following exposure to ethanol on various schedules. Psychopharmacologia, 29, 203–210. 4702273 10.1007/BF00414034

[brb3523-bib-0064] Wise, R. A. (2013). Dual roles of dopamine in food and drug seeking: The drive‐reward paradox. Biological Psychiatry, 73, 819–826. 23044182 10.1016/j.biopsych.2012.09.001 PMC3548035

[brb3523-bib-0065] Wise, R. A. , & Bozarth, M. A. (1987). A psychomotor stimulant theory of addiction. Psychological Review, 94, 469–492. 3317472

[brb3523-bib-0066] Wise, R. A. , & Koob, G. F. (2014). The development and maintenance of drug addiction. Neuropsychopharmacology, 39, 254–262. 24121188 10.1038/npp.2013.261 PMC3870778

[brb3523-bib-0067] Wise, R. A. , & Rompre, P. P. (1989). Brain dopamine and reward. Annual Review of Psychology, 40, 191–225. 10.1146/annurev.ps.40.020189.001203 2648975

[brb3523-bib-0068] Xuei, X. , Dick, D. , Flury‐Wetherill, L. , Tian, H. J. , Agrawal, A. , Bierut, L. , … Edenberg, H. J. (2006). Association of the kappa‐opioid system with alcohol dependence. Molecular Psychiatry, 11, 1016–1024. 16924269 10.1038/sj.mp.4001882

